# Not ready in the ways that count– a qualitative exploration of junior doctor’s perceived preparedness for practice using Legitimation Code Theory

**DOI:** 10.1007/s10459-024-10380-w

**Published:** 2024-10-07

**Authors:** Stuart Redvers Pattinson, Hans Savelberg, Anique Atherley

**Affiliations:** 1 Unit for Undergraduate Medical Education (UUME), School of Clinical Medicine, Faculty of Health Sciences, University of the Witwatersrand, Johannesburg, Gauteng, South Africa; 2 School of Health Professions Education (SHE), Maastricht University, Maastricht, Netherlands; 3 Department of Nutrition and Movement Science, School of Health Professions Education (SHE), Faculty of Health, Medicine and Life Sciences, Maastricht University, Maastricht, Netherlands; 4 Office of External Affairs, Ross University School of Medicine, Bridgetown, Barbados

**Keywords:** Transition from student to doctor, Legitimation code theory, Undergraduate medical education, Preparedness for practice

## Abstract

Despite demonstrating the required competencies to graduate, many newly qualified doctors find the transition to internship difficult. There is a concern over whether their preparation is aligned with the expectations of the role. This study aimed to gain a better understanding of the competencies needed for legitimate practice as junior doctors and explores their perceived preparedness for practice. A qualitative, descriptive study using focus groups was undertaken with first year internship doctors. Thirty-two junior doctors in their first year of internship took part in five focus groups. The data were analysed using a reflective thematic analysis approach with a subsequent analysis using the Legitimation Code Theory (LCT) specialisation dimension coding framework to aid interpretation. Personal attributes including adaptability, organisation and proactivity form the basis of achievement in internship. While graduates felt ready in some ways, it was not in the ways that counted. Participants felt well prepared in terms of their clinical knowledge and skills, but legitimacy came from being able to take responsibility, communicate effectively and apply knowledge confidently and efficiently to all aspects of patient care, something that they did not feel ready to do. Using LCT has revealed a shift in the basis of achievement between medical school, where individual academic performance is rewarded, and internship, where personal and social competencies are legitimised. There is a clash between what graduates feel well prepared for and the expectations and demands of the internship role, resulting in a difficult and stressful transition from student to doctor.

**Supplementary Information:**

The online version contains supplementary material available at 10.1007/s10459-024-10380-w.

## Introduction

One goal of undergraduate medical education is to prepare students to make the challenging transition from student to doctor (Burford & Vance, 2014). In South Africa, newly graduated doctors must complete a mandatory, two-year internship at an accredited public hospital after they graduate from medical school. Challenging transitions such as this can be a stimulus to learning, enabling increased competency and confidence as well as insights about themselves and their work (Carlsson et al., 2023), but only if they are manageable (Sturman et al., 2017). If a graduate has been well prepared for the transition they have the potential to thrive, experiencing a sense of being able to acquire and apply knowledge and skills, leading to feelings of wellbeing, productivity, achievement and competency in job performance (Niessen et al., 2012). A lack of preparedness, however, can make this transition abrupt, stressful and overwhelming, resulting in anxiety, difficulty in maintaining self-care and social relationships and reduced self-confidence (Carlsson et al., 2023; Sturman et al., 2017). This potentially leads to burnout which can impact internship doctor (intern) wellbeing, patient care and the retention and recruitment of doctors (Brennan et al., 2010; Dornan et al., 2020; Hariharan & Griffin, 2019; Liebenberg et al., 2018). Considering that high levels of stress (Sun et al., 2008), depression (Naidu et al., 2019) and burnout (Dubale et al., 2019) have been found among South African interns, there is a concern that graduates may not be well prepared to thrive in their new role.

For an individual to thrive in a given setting they need to demonstrate *legitimacy* — displaying the knowledge and ways of being which are valued and considered the *basis of achievement* in that context (Maton & Tsai-Hung Chen, 2020). Legitimacy involves understanding the underlying principles and ‘rules of the game’ that allow for the execution of a role in a way that is valued and rewarded by the culture and community in which one practices. To assess whether students are being adequately prepared for their new role as a junior doctor, a clear understanding of the competencies they need in order to succeed and thrive in the role is important. Our current understanding has been limited by a leaning of the literature on ‘preparedness for practice’ to focus on clinical knowledge and skill proficiencies as indicators of preparedness (de Villiers et al., 2018). This has left a gap in our understanding regarding the social, personal and professional competencies needed for a successful transition (Atherley et al., 2019). Successful graduation already suggests that adequate competency in clinical knowledge and skills have been demonstrated in order to meet requirements (de Villiers et al., 2018), so it is worth investigating if it is in other areas that students could be better equipped. This study aimed to make explicit the competencies in various domains (knowledge, skills, attitudes and ways of being) that make up the basis of achievement, and therefore legitimacy, for junior doctors in their particular context and explores their perceptions of how well they were prepared to succeed in this role.

Research in the South African context is important because the majority of studies done on this transition from undergraduate to junior doctor have taken place in the Global North, which may have different clinical contexts and expectations of junior doctors, potentially limiting the transferability of their findings (Cameron et al., 2014). Frambach et al. (2015) raise a concern over the underrepresentation of countries in sub-Saharan Africa in medical education research, highlighting the need for evidence within this context to encourage innovation and overcome resistance to change. They describe challenges faced by junior doctors in this region as being “of a different dimension altogether” (Frambach et al., 2015, p.71). This is a region that could potentially benefit the most from curriculum innovation where curricula face the challenge of innovation focused on the reality of local health care needs while being connected to global ideals (van Heerden, 2013).

South Africa has a patient population with a high burden of trauma and infectious diseases and internship doctors must be prepared to face high work and patient loads, long hours, limited resources and demanding levels of decision making and responsibility for patients’ lives (Jaschinski & De Villiers, 2008; Kent & de Villiers, 2007). While the intention of internship is to gradually prepare interns for independent practice through exposure to the realities of clinical medicine under a degree of supervision (Jaschinski & De Villiers, 2008), it is frequently the case that supervision for interns in South Africa is limited or unavailable (Bola et al., 2015). As a result, the “first year as a doctor may be a stressful, overworked and poorly-supported experience” (Jaschinski & De Villiers, 2008, p.70a). The reality is that many of these local demands are unlikely to change in the immediate future, and while improved infrastructure and numbers of healthcare workers would obviously help they are not solutions within the scope of medical education (Frambach et al., 2015). The transition will always be challenging, from an undergraduate curriculum perspective the task is to focus on what can be changed to make best use of available learning opportunities to ready graduates to succeed in overcoming these challenges and thrive. It is critically important for research to be conducted in this context to understand if there are specific ways in which students need to be prepared to face these particular challenges that may not be understood from the current literature.

Regularly gaining graduate perceptions about their preparedness for the workplace is an important and powerful tool in curriculum evaluation (Jaschinski & De Villiers, 2008). Awareness of the resources needed to successfully navigate this complex transition within a local, Global South, context is essential in curriculum planning going forward (Gordon et al., 2017). Curriculum innovation that improves student’s preparedness will positively impact the personal and work lives of junior doctors (Cameron et al., 2014).

## Study Aim

The aim of this study was to answer the following research questions:

1. What competencies form the basis of achievement in the internship setting and are needed for legitimate practice?
2. How well prepared do medical graduates perceive they are for the internship role?

These answers will allow for undergraduate curriculum evaluation in terms whether the preparation of students is aligned with the reality and requirements of the role, potentially revealing ways in which undergraduate curriculum could better prepare medical graduates for their responsibilities during internship. This research aims to identify opportunities for meaningful innovations that will make the most of limited resources to better equip all students to thrive as interns. This study builds on the work of Atherley et al. (2019) in considering the non-educational aspects of the transition in a Global South context and responds to the call for research on this topic in the South African setting (Cameron et al., 2014; Frambach et al., 2015). This research adds the South African interns’ perspective to the supervisor perspective studied by de Villiers et al. (2018).

## Methods

### Study design

This qualitative, descriptive study is grounded in a Social Realist Paradigm (Maton, 2014), which is concerned with both knowledge and knowers, exploring how knowledge is shaped and valued within contexts. Social realists argue that while knowledge is socially produced, it does not follow that all knowledge is relatively equal (Carver, 2020). This study aims to holistically conceptualise the basis of achievement in the internship context and, therefore, requires the application of a paradigm that is neither blind to the potential significance of the personal attributes of knowers (which has been the case with many ‘preparedness for practice’ studies which have tended to focus mostly on knowledge and skills), nor blind to the fact that having certain types of specialised knowledge and skills may be valued or legitimised over others in the health professions. Focus groups were used to gather data as they allow for conversational exchanges that provide insights into what participants are thinking and feeling and the circumstances in which they construct these meanings and are a method of choice for program evaluation (Stalmeijer et al., 2014).

### Theoretical framework

Legitimation Code Theory (LCT) provides a powerful theoretical framework for understanding how knowledge and ways of knowing are valued in different contexts by analysing actors’ dispositions (in this case interns), and the organising principles of their practices (Maton & Tsai-Hung Chen, 2020).

LCT comprises three active dimensions currently being used in educational research: Specialisation, Semantics and Autonomy. Each dimension offers a lens through which different aspects of educational practices can be explored. *Specialisation* focuses on the basis of achievement underlying practices in various contexts, helping to understand what types of knowledge and personal attributes (knowledge-knower relationships) are valued and legitimatised. *Semantics* explores the complexity and context-dependence of practices. *Autonomy* looks at the relationships between different sets of practices, like knowledge from different disciplines. *The semantics* dimension of LCT has been previously used in medical education research into critical reflection (Monbec et al., 2020) which led to a revised analytical rubric which makes visible what is valued in nursing reflection tasks, as well as in problem-based learning (PBL) (Hassan, 2020), showing the need to adapt and contextualise PBL when implementing it across different disciplines. Maton (2014) recommends only using as much theory as the problem demands and Specialisation was selected for this study due to its alignment with the research aims.

*Specialisation* is especially relevant for this study as it provides a toolkit for analysing what kinds of knowledge (epistemic relations) and personal attributes or ways of knowing (social relations) are legitimised within the internship context. By exploring these factors, this study hopes to reveal what is considered legitimate knowledge and who is recognised as a legitimate knower in the clinical setting. Using *specialisation* allows the study to compare the value placed on formal medical knowledge and procedural skills with the value placed on interns’ personal attributes. This lens can make explicit the ‘rules of the game’ within the South African clinical context, which might be transferrable to other Global South contexts. The use of the Specialisation dimension allows for the basis of achievement within the internship to be revealed by exploring interns’ perceptions and experiences and making the underlying messages they receive about what is rewarded and viewed as success within that context explicit.

The *specialisation* dimension is applied in qualitative research by analysing themes, identified through a thematic analysis, based on the relative strength (+) or weakness (-) of their epistemic and social relations. This then allows themes to be coded into one of four codes:

- Knowledge codes (ER+, SR-): emphasis on specialised knowledge over personal attributes (what you know) as the basis of legitimacy.
- Knower codes (ER-, SR+): emphasis on personal attributes over specialised knowledge (who you are) as the basis of legitimacy.
- Elite codes (ER+, SR+): importance placed on both specialised knowledge and personal attributes related to the context; possessing both specialised knowledge and being the right kind of knower is the basis of legitimacy.
- Relativist codes: (ER-, SR-) where neither specialised knowledge nor personal attributes are emphasised and anything goes.

**Fig. 1:**
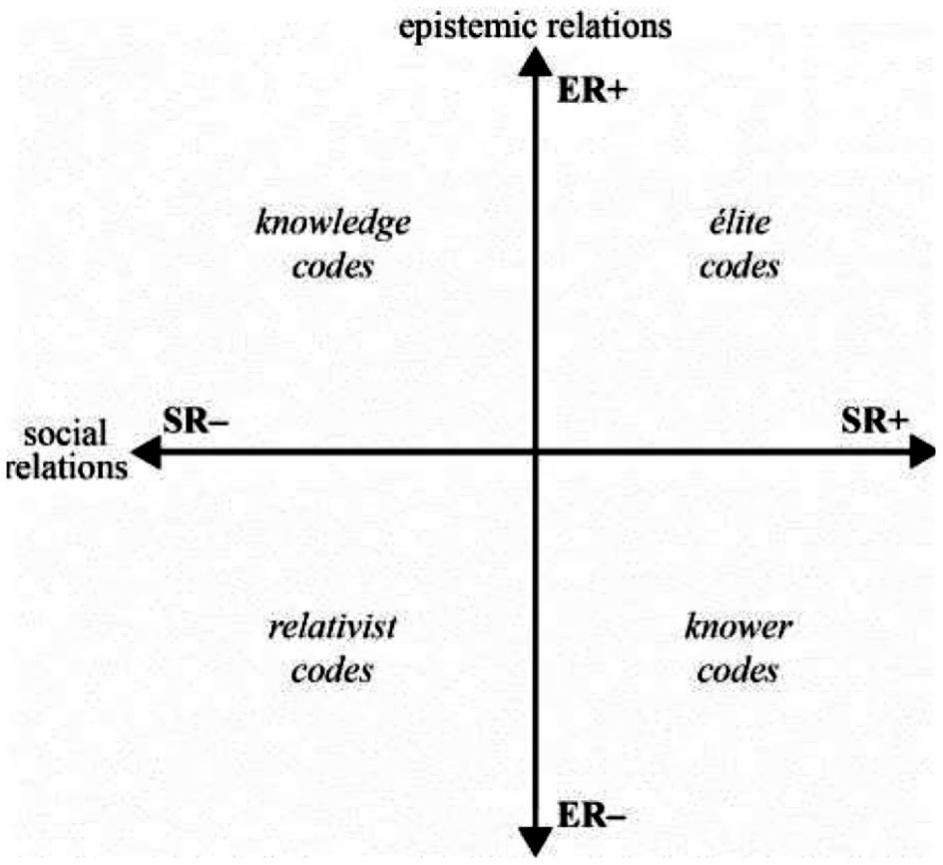
The specialization plane (Maton, 2014, pg 30)

Codes in LCT help to identify the basis of achievement or legitimacy in a given context. As initial codes are generated in a thematic analysis, they are assigned strengths and weaknesses of epistemic and social relations using a translation device (Table 1). The translation device links strengths and weaknesses of epistemic and social relations with indicators of their manifestation in the dataset (Maton & Tsai-Hung Chen, 2020). As themes are then generated, reviewed and refined they are coded as either knowledge, knower, elite or relativist codes based on an overall picture of what is legitimised and forms the basis of achievement within that theme. This is a qualitative process based on discussions within the research team of the relative overall strengths and weakness of epistemic and social relations within an identified theme. The findings are illustrated by plotting themes on a Specialisation Cartesian plane created by two axes that represent the continua of strong to weak epistemic and social relations; see Fig. 1. When assessing relations, the researchers may for example, place a theme emphasising the need to master a specific skill but downplays personal attributes as it relates to that skill as having strong epistemic relations (focus on knowledge/skill related to that procedure) and weak social relations, falling into the knowledge code quadrant. This process reveals an overall visual representation of the “legitimate basis of practices, beliefs and identity” (Maton, 2014, p.12); making explicit what is legitimised in that setting, in our case, internship.

**Table 1:** Translation device

Epistemic and Social Relations	Indicator	Example
ER +	Content knowledge and clinical skills emphasised as the basis of achievement	*“I think academics and knowledge were definitely the most valuable”*
ER -	Content knowledge and clinical skills downplayed	*“But I wouldn’t say it’s really like anything too drastic in terms of like intelligence or skills”*
SR +	Personal attributes and social skills form the basis of legitimate practice	*“It’s mainly like communication*,* organization and then just being adaptable as well”*
SR -	Personal and social attributes not valued or rewarded	*“I didn’t expect so much of the emphasis to be on working as a team. I don’t know if that emphasis is placed as much on us as students”*

The placement of themes on the specialisation plane assists researchers to unpack the underlying ‘rules of the game’ within the internship context— what is valued, who is recognised as successful and why. This analysis uncovers code clashes (conflicts between different codes in a single context such as internship) and code shifts (the basis of legitimacy and achievement changes between different contexts (e.g. internship vs. medical school) and allows their impact to be explored. Identifying code clashes and code shifts can be valuable in understanding why certain individuals may struggle within a particular context. If, for example, their personal attributes clash with the behaviours that are valued or there is a shift between what is legitimised at medical school and the internship settings, then successful graduates may find themselves struggling as they navigate the challenge of figuring out how to behave in a way that is valued. Understanding these dynamics can offer valuable insights for improving educational practices and supporting interns as they transition from medical students to practicing doctors.

### Context

The medical degree at the University of the Witwatersrand (Wits) in Johannesburg, South Africa, is a six-year degree with a graduate entry programme into the third year of study for applicants who have completed a relevant prerequisite degree. The first four years of the degree are largely medical school based and the final two years consist of a clinical rotation based clerkship in health care facilities affiliated with the university. Graduates must then complete a mandatory, two-year internship at an accredited training facility. Internship placements are available in all nine provinces in South Africa and are allocated by the Department of Health after an application process.

### Study population

First year medical interns, graduated from the University of the Witwatersrand, were purposefully sampled (Reeves et al., 2013) to take part in the study. This was based on convenience and the variation of internship experiences they would be able to share, as graduates are placed at multiple different locations across the country. During the final month of their studies, all final year medical students were emailed via official university channels with information about the study and asked to indicate if they would consent to being contacted regarding the study once they had begun their internship. In total, forty of the 340 students invited indicated their willingness to be contacted and provided contact information. They were all then emailed three months into their internship and invited to take part in a focus group and were given opportunity to choose from a number of possible dates. All willing and available interns were included, resulting in 32 interns (17 women and 15 men) from a wide range of institutions around the country taking part in five focus groups ranging from 5 to 7 participants each.

### Data collection

Focus groups were conducted online in April 2023 via *Microsoft Teams* and were based on a semi-structured interview guide (Annexure A) that was developed by the researchers based on the research questions and the relevant literature. The online platform allowed the focus groups to be recorded and for participants to join from various locations across the country. Participants in the focus groups agreed to maintain the confidentiality of the discussions. The principal researcher (SP) moderated the focus groups. An observer (AA) was present for the first focus group to take notes and give feedback regarding the moderation to the principal researcher (SP). Based on the observations and feedback, SP then facilitated the remaining focus groups independently.

### Data analysis

The data from the focus groups were transcribed by the *Microsoft Teams* software. This was checked and edited against the audio recordings and pseudonymised by the principal researcher (SP). Each participant was randomly assigned a research number. The transcripts were then uploaded onto *Atlas.ti* qualitative analysis software to undergo a reflexive thematic analysis based on Braun and Clarke’s suggested criteria (Braun & Clarke, 2021; Kiger & Varpio, 2020). The authors familiarised themselves with the data and then generated initial codes. As these codes were generated, they were assigned strengths and weaknesses of epistemic and social relations based on a translation device devised by the authors in an iterative process alongside the data analysis (Table 1). The translation device explains how different strengths manifest in the dataset and gives an illustrative example, allowing the initial codes to be further coded according to the LCT Specialisation dimension coding framework. Initial themes were generated which were then reviewed and refined before being defined and named. Each of these themes were coded into knowledge, knower, elite and relativist codes based on the strength and weakness of the epistemic and social relations of the initial codes from which the theme was developed.

### Ethical considerations

This study was approved by the University of the Witwatersrand Human Research Ethics Committee (Protocol number M221030) as well as the Maastricht University Faculty of Health, Medicine and Life Sciences Research Ethics Committee (Approval Number: FHML-REC/2023/009). Written informed consent was obtained from all participants prior to the focus groups. It was stressed that participation was voluntary and that the participants could, at any time, withdraw their participation.

### Rigor

The number of focus groups was limited by the number of available participants but generated enough information power (Malterud et al., 2016) to meaningfully answer the research question. This assessment was based on the dimensions of: the broad study aim, the specific sample of first year interns from one university, the use of established theory (LCT), high dialogue quality and the thematic analysis strategy (Varpio et al., 2017). The initial coding of focus groups one and two was done by SP and AA independently of each other. They then met to develop an initial coding framework together. SP then coded the final three focus groups and generated the initial themes, which were discussed with the whole team to reach an agreement on the final themes.

### Reflexivity

The research team consisted of a clinical lecturer pursuing a PhD in Health Professions Education (SP) and two experienced educationalists and researchers (AA and HS). The principal researcher (SP), who teaches in the students’ third and fourth years of study, came to this project out of a concern for a pervasive sense of dread he began to notice in undergraduate students concerning the internship years that lay a few years ahead. He then started to hear from alumni who were really struggling with their internships despite having been very successful as students, increasing his motivation to explore whether the undergraduate curriculum was possibly missing something or could be doing more to ready graduates for a successful transition. HS is a developer of education in the health professions domain and teaches in bachelor and master programmes in Health Science, Biomedical Sciences and Medicine. As a teacher and curriculum developer, he noticed the challenge to develop programmes that drive students’ intrinsic motivation to study. As an educational scientist, he focuses on understanding design components that facilitate intrinsic motivation; feeling prepared and competent is one of the determinants of intrinsic motivation. In this context, being qualified to perform the medical treatments is not sufficient; competencies in the social and personal domain are also required. AA is a medical doctor, health professions educator and teacher within a medical school (community medicine focus) and postgraduate programmes (health professions education focus). She has a research interest in transition periods across the medical continuum and has conducted research in this area, which are published. She has worked and conducted research internationally and with diverse participant populations. She brought her own knowledge and biases to this work, which was discussed extensively during team discussions.

The principal researcher (SP) was familiar to the participants, as one of their prior educators in their pre-clinical years of study, but had no involvement, influence or authority over their teaching, work, assessment or progress as final year students or interns. Being known in the context gave the researcher access to the participants but may have affected who volunteered, based on their previous experience of the researcher as an educator. It may have also affected how critical they were willing to be of the undergraduate curriculum or how openly they felt they could share their shortcomings or struggles. The rest of the research team were both outsiders to the research context and had no previous contact with the participants.

## Results

Two key findings, relating to the two research questions, were identified from the data through the lens of LCT. First, personal attributes– ‘who you are’ - determines legitimacy as an intern in this context. Second, despite graduates feeling prepared in many respects, it was not in the ways that counted the most within the internship context.

### Finding 1: who you are determines legitimacy as an intern

Personal attributes with stronger social relations (SR+) — willingness to learn, adaptability, organisation, efficiency and teamwork — were identified as key factors in what makes a ‘good’ intern. Adaptability and a willingness to learn were identified by participants as some of the most valuable of these attributes, as they were important when dealing with the steep learning curves associated with inhabiting a role that was constantly changing as described by Participant 2:

I’d just emphasize the adaptability.…A lot of times you’re getting exposed to new environments and new roles, like, on almost a daily basis. And so for that reason, I think you need to have, like, a strong head on your shoulders… in other words, just like, not be too afraid to take on those new roles. (SR+) (P2, Focus Group 1).

Participants described interns as integral to the functioning of clinical teams, primarily tasked with carrying out the plans of the senior team members. In this role, interns’ clinical knowledge and skills were often downplayed, with the expectation that they simply demonstrate “*basic competency” (P29*,* Focus Group 1)* (weaker epistemic relation; ER-). Instead, personal attributes such as their organisational acumen, time management, efficiency, work ethic, proactivity and ability to work within an inter-professional team was highly valued (stronger social relations; SR+). As one participant summarised:

But I wouldn’t say it’s really like anything too drastic in terms of like intelligence or skills. It’s mainly like communication, organization and then just being adaptable as well.…Even if you’re not a really good clinician in your diagnosis or skills, if you just able to identify what needs to be done, when it has to be done and organize when you’re going to do it, that’s probably the most helpful skill. (ER-, SR+) (P6, Focus Group 1).

Participants highlighted that interns could be de-legitimised if perceived to be ‘not pulling their weight in the team’, requiring others to compensate for their lack of efficiency. Efficiency and organisation was seen as fundamental for success, the basis of achievement, in the clinical team (strong social relations; SR+). Interns who took a long time to do their workload were viewed as less valuable.

Participants found the intern role hard to define because *“people have different ideas of what an intern is supposed to do” (P22*,* Focus Group 1)*. The role differed across departments, between different environments (such as wards, clinics and theatre) and at different times of day; particularly between regular hours and after-hour calls. Participants described interns as “*spot fillers” (P29*,* Focus Group 1)*, expected to execute whatever task needed doing at a given moment:

it seems like the role of the intern is kind of to do everything, you know, like you have to be able to do everything and anything (ER+, SR+) (P20, Focus Group 4).

This dynamic role necessitated interns who were adaptable, hardworking and eager to learn (stronger social relations; SR+). These personal attributes enabled them to manage change and acquire the skills needed for each new task while on the job, establishing their legitimacy in each new context.

In order to be better prepared for internship, the participants suggested that students need to be more proactive and self-directed, make the most of clinical learning opportunities, learn to work in a team and actively work on developing the skills that will make them ‘good’ interns. Their attitude towards learning and the type of learner they are makes all the difference.

the kind of student I think you were directly translates into how seamless your transition into internship is.…I don’t even think it’s so much the emphasis on books. I think it’s just like, how much did you apply yourself in the clinical space? (ER-, SR+) (P10, Focus Group 2).

Overall, findings suggest that the basis of achievement in the internship context aligns with a knower code (ER-, SR+) where interns are primarily valued for their personal attributes — such as being adaptable, organised, efficient, and willing to learn — rather than for possessing specialised knowledge.

### Findings 2: interns were not ready in the ways that counted

The transition from student to doctor was challenging for most participants, revealing a gap in their preparedness and the legitimacy required in the internship context. Despite varying degrees of personal resilience, the primary emotions during this period were largely negative — fear, uncertainty, stress, anxiety, disappointment, frustration and being overwhelmed. This sense of unpreparedness led to feelings of inadequacy as they began internship and illustrated a mismatch between forms of legitimacy expected in practice and what participants felt capable of at the outset as described by participant 2.

starting out I felt quite useless and I’m sure a lot of us had those feelings (P2, Focus Group 1).

Readiness varied for different intern role requirements. Participants generally felt well prepared in terms of the “*theoretical domain*” *(P22*,* Focus Group 1)*– “*clerking*,* histories*,* exams and procedures” (P3*,* Focus Group 1)*, which reflects stronger epistemic relations (ER+). They noted that this aspect of their preparation was not a big shift from their experience as students. In contrast, what participants felt least prepared for as they started internship was the sudden and significant increase in the responsibility for patient care. The level of supervision and support varied significantly, ranging from heavily supervised patient care to complete independence, differing across facilities, departments and times of day as described by Participant 16:

I just want to emphasize that during the day or in the morning, you are the most junior member, but at night you’ll be the most senior (P16, Focus Group 3).

The heightened accountability and decision making regarding patient care, particularly in emergencies where they were often the first doctor on the scene in the ward, was a stark shift from being a student:

It was definitely an adjustment to kind of get used to having all the responsibility of, like, you know, where you are the doctor, you are the one kind of making decisions, you are responsible for your patient’s care. Whereas, as a student is not so much the case, where you will just pop in, you’ll do a few things here and there, you’ll go to your tut and you’ll leave, whatever the case is. Here you are like, the guy (ER+, SR+) (P19, Focus Group 4).

To address these challenges, interns needed to exhibit both strong knowledge and clinical skills (stronger epistemic relations; ER+) *and* also the personal attributes of confidence and initiative (stronger social relations; SR+). Participants generally felt confident in seeing patients and creating management plans but lacked the practical know-how they needed actually carry out the plan, and do so under time pressure. Interns were expected to be independent while knowing their limitations and when to ask for help. Participant 21 reflected on the difficulty of adjusting to this shift in responsibility:

that, for me anyway, was just, like, a hurdle because, you know, you always knew in theory, you’d always be tested on it and you’d get the questions right. But now it’s like, okay, you know what you need to do, but now you need to write it down and put your signature next to it, that you’re going to be doing this to a patient. And, you know, that’s scary because, what if something happens? (ER+, SR+) (P21, Focus Group 4).

Comprehensive patient care also required excellent teamwork and communication skills with patients and the interprofessional team. Participants were surprised by the emphasis on teamwork, which was not highlighted as critical during their time as students as described by participant 11:

I didn’t expect so much of the emphasis to be on working as a team. I don’t know if that emphasis is placed as much on us as students because there wasn’t so much responsibility (SR-) (P11, Focus Group 2).

Written and verbal communication proved to be a challenge for participants. This ranged from discharge summaries and advocating for patient care, to the daunting task of consulting with other disciplines. This ability to synthesise and communicate patient information requires both knowing what information needs to be conveyed (ER+) and how to communicate it effectively (SR+). Participant 24 highlighted just how critical a skill communication is in successfully carrying out their job:

Even if you have all the knowledge, if you’re not really confident, then it doesn’t really translate very well. Even if you’re really good at managing your time, if you struggle to speak to your senior, speak to people from other departments, speak to allied services, then it doesn’t really translate, and then it becomes very difficult to do your job. Even interacting with patients. It just becomes very difficult to establish yourself. (ER+, SR+) (P24, Focus Group 4).

Overall participants felt well prepared in terms of their clinical knowledge and skills, but struggled with decision-making, taking responsibility and communication. Just having knowledge was not valued. This mismatch indicates an elite code where knowledge is important (ER+) but only when it is enacted by right kind of knower (SR+). Legitimacy came from being able to apply knowledge confidently, efficiently and in a team, something they were generally not ready to do.

In order to be better prepared for legitimate practice as interns, participants suggested they needed more clinical exposure, earlier in their studies, in order to gain the practical experienced needed to be more confident and hands-on later on as senior students. Once in their senior clerkships, they needed to be actively involved in patient care as legitimate members of clinical teams. There was a perception among participants that, as students, they were not always a priority, often feeling like visitors in the clinical setting. They needed defined roles and a greater expectation on them to take responsibility for compete patient care:

if we could emphasize the student’s role a bit more in terms of start to finish patient management. So giving students a patient to manage from admission, to work up in the wards, to discharge or whatever. You become a lot more nuanced and aware of the whole process. (ER+, SR+) (P11, Focus Group 2).

This would not only encourage the development of medical expertise, but also allow for professional development through meaningful application of that knowledge and enculturation into the field, learning the ‘rules of the game’ that make them legitimate knowers.

Participants suggested that the medical school needs to communicate clearly to the students what competencies they will need develop for legitimate practice as interns and demonstrate that it values these through its assessment practices. For the participants it was clear that individual theoretical knowledge was what counted when they were in medical school, leading to them seeking out formal teaching and study time over work place based learning and hands-on patient care as part of a clinical team:

I think less needs to be put on tests and examinations and more on trying to get that experience from the students because I think what’s happening is; the students are too stressed about their exams and the tests and are not really spending enough time in the hospitals and we’d rather dodge, duck and dive to study than to actually be in the space where they will be in the next two years and or in the next year. And I think that’s that exposure that we need. (ER+, SR-) (P29, Focus Group 1).

**Fig. 2:**
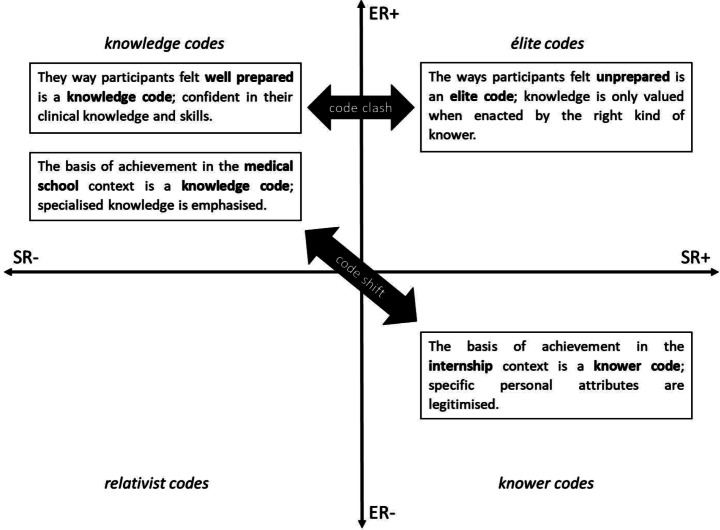
Summary of results on the specialisation plane

The perceptions of what learning the university values represent a knowledge code (ER+, SR-), in contrast to the learning in the form of elite and knower codes that they identified would have been most valuable in readying them for internship. The overall results are summarised in Fig. 2 below.

There is a shift in the basis of achievement between medical school, where individual academic performance is rewarded (a knowledge code) and internship, where personal and social competencies such as adaptability, organisation, efficiency and teamwork are valued (a knower code).

There is a clash between the ways participants felt well prepared (clinical knowledge and basic skills - knowledge codes), the ways they felt they needed to be well prepared for legitimate practice. They did not feel ready for decision-making, taking responsibility and interpersonal communication (elite codes).

## Discussion

This study used the LCT Specialisation coding framework to explore the interns’ perceptions of what competencies are legitimised in the clinical setting and how ready they were for the role. Our results highlighted a clash between how interns felt prepared and the competencies required to be an effective intern.

*Who you are* provides legitimacy in internship. Personal attributes of adaptability, readiness to learn, organisation, efficiency, proactivity, confidence and teamwork formed the basis of achievement in this internship context. However, many participants in this study felt unprepared in these areas. They also struggled to apply their knowledge to patient care, leading to challenges in taking responsibility, making decisions, communication and handling emergencies. These difficulties led to fear, stress and feelings of inadequacy. This is important because subjective unpreparedness (Dornan et al., 2020), a lack of confidence in one’s ability to apply theory to practice and a lack of adaptability (Hariharan & Griffin, 2019) all predict poor well-being and burnout.

Individual academic performance formed the basis of achievement during medical school; meaning there is a shift from valuing ‘what you know’ to ‘who you are’ between the undergraduate and internship contexts. Interns began this job expecting that they were adequately trained with the wide variety of clinical, personal and social competencies required for successful practice, but quickly realised there was more to accomplish, resulting in an abrupt change from “self-confidence to self-doubt and uncertainty” (Sturman et al., 2017, p3) with profound implications on their ability to cope. This clash between what they are good at and what forms the basis of achievement in internship setting led to difficulty adapting to new expectations and a challenging period during which they were trying to figure out the ‘rules of the game’ and how to behave in different ways that are valued. This kind of code clash can render previously successful students unable to continue to succeed (Maton & Tsai-Hung Chen, 2020).

The attributes legitimised in the South African context align with those described by what South African intern supervisors look for in a ‘good’ intern de Villiers et al. (2018). International studies have shown that medical graduates often lack preparedness in administrative and management skills, communication skills, practical ‘know-how’ and coping skills which are important in clinical settings (Sturman et al., 2017) and help medical trainees learn in new environments (Teunissen & Westerman, 2011b). While the need for reflexivity, responsibility and resilience, and to develop proactive and self-regulatory learning strategies, have been emphasised in other contexts (Padley et al., 2021), our study reveals the high stress that results from a lack of preparedness within a setting where new junior doctors are expected to be independent, make high stakes decisions, manage emergencies and take accountability for patient care. In South Africa, newly graduated doctors need to be ready to for instances, from early on, where they will work unsupervised and therefore should go into their internships prepared with the confidence to take on that responsibility. They need more than just knowledge and skills; they need to know how to do the job. Our findings show the gaps in preparation that come from students not being fully involved in patient care or only doing part tasks, with our participants struggling to apply knowledge and carry out comprehensive management plans. Dornan et al. (2020) aptly describe unpreparedness as “knowing what but not how” (Dornan et al., 2020, p.1).

In a resource-constrained setting, efficiency is highly valued as there are very few doctors to see many patients. Students who performed well during medical school, where demonstrations of extensive knowledge was rewarded, may struggle if they are perceived as ‘slow’ as interns, finding themselves delegitimised. The disconnect between medical school training and internship expectations showcases that medical students should be integrated into clinical teams early to learn team dynamics and understand pressures they need to be prepared for in the South African context.

In much of the Global North, patient safety guidelines are a major reason students have limited opportunity to practice in the clinical setting (Brennan et al., 2010; Teunissen & Westerman, 2011a). In our context, it was primarily a lack of constructive alignment (Biggs & Tang, 2013) between the competencies expected of interns once they enter practice and those valued in students during their clerkships that led students to underutilise opportunities to be hands-on with patients. Students were torn between excelling in academic assessment that rewarded individual demonstrations of knowledge and skills and spending time in the clinical setting gaining workplace-based experience and practical know-how (Sturman et al., 2017). LCT shines light on the fact that that students are not motivated to develop the personal attributes needed to succeed in internship, despite having the opportunity. As a result, students are graduating without the skills that would most improve their chances of thriving during internship.

LCT takes our knowledge of what is required of a newly qualified junior doctor beyond just a description of the practical requirements of the job to a much deeper understanding of what makes an individual successful at the role and valued as a member of the clinical team. This imparts a significant responsibility onto curriculum designers to ensure that these key competencies are built into learning opportunities and assessments and valued throughout their undergraduate career. LCT makes explicit that the ways in which students are currently being prepared for internship and the ways in which they need to be prepared are not aligned; this has significant consequences for medical trainees’ wellbeing and success. Curriculum re-evaluation, focussing on a culture change about the role of students in the clinical space, is needed if future graduates are going to be better equipped to thrive, rather than just survive, during internship.

### Recommendations for future practice

The need for learning objectives that encompass a variety of competencies in various domains beyond just medical knowledge has been recognised before and is reflected in well-known competency frameworks such as CanMEDS (Frank et al., 2015). Such frameworks are only impactful, however, if they are made explicit and are meaningfully translated into curriculum through constructively aligned learning activities and assessments (Biggs & Tang, 2013). There has been a tendency, however, in medical education to focus just on one educational purpose– qualification (based on demonstrations knowledge and skills), rather than on socialisation of students to the ways of being that are legitimatised in the clinical setting or the development of individual student’s personal agency, resilience and responsibility (Biesta & van Braak, 2020). In order realise these wider educational goals, students need more and earlier clinical exposure (Monrouxe et al., 2018) where they are be actively involved in patient care as legitimate members of clinical teams with defined roles. Learning in the clinical space only becomes meaningful when students start to feel useful (Sturman et al., 2017). Assessment programmes need to show that they value the development of personal, social and professional competencies, along with medical expertise, through carefully considered workplace based assessments (Pangaro & Ten Cate, 2013). Improved preparedness resulting from these curriculum innovations will make a big difference in reducing burnout and improving the wellbeing of junior doctors (Stodel & Stewart-Smith, 2011).

### Limitations and recommendations for further studies

While this study involved interns from a single institution, which could limit transferability, the findings were consistent with studies done both elsewhere in South Africa (de Villiers et al., 2018) and overseas (Dornan et al., 2020; Sturman et al., 2017; Teunissen & Westerman, 2011a) suggesting that they have relevance for other contexts where students have to make a similar transition to practice. The moderator of the focus groups (SP), is known to the participants as one of their previous educators, which may have introduced bias affecting participants’ openness and willingness to be candid about their struggles (Stalmeijer et al., 2014). The moderator may also have influenced the discussions based on their own familiarity with and experience of the course and clinical setting.

While the use of synchronous online focus groups allowed special and temporal barriers to be overcome as interns could join from multiple distant geographical locations at times that suited them after work hours, they do have some potential limitations. Online focus groups require sufficient access to technology, data and connectivity and online communication can limit participants’ experience of social cohesion and communication through gestures, non-verbal cues and eye contact. Participants may demonstrate less turn-taking and shorter responses in online focus groups but these differences tend to decrease over time and research suggests no substantial differences in data quality between online and in-person focus groups (Keemink et al., 2022).

This study explored interns’ perceptions, and while this retrospective data is very valuable, correlation with actual performance is sometimes uncertain (Bleakley & Brennan, 2011). Goodson & Vassar in 2011 (2011) called for additional perspectives regarding the transition to doctor using observational and longitudinal research designs to provide a better and broader understanding of the factors impacting students’ ability to successfully transition to internship, yet there is still paucity of studies in the South African context using these methodologies. Our future work will answer this call.

This study did not explore how the intersections of race, gender and sexual orientation impact the transition from student to doctor. Mokhachane et al. (2023) showed how norms and values based on Western concepts of professionalism can result in certain groups being legitimised while others are marginalised. Considering South Africa’s history of Apartheid and racial inequality, it would be valuable to explore the role that factors such as race may have on preparedness for practice and legitimacy in the clinical setting.

## Conclusion

There is a clash between what graduates feel well prepared for and the expectations and demands of the internship role, resulting in a difficult and stressful transition from student to doctor. Participants recognised the personal and social competencies required for legitimate practice, but also highlighted how the lack of value placed on these in the undergraduate space results in graduates well prepared in terms of knowledge and skills but not in the ways of being that form the basis of achievement in the internship setting. Clinical knowledge is only valuable if it can be applied confidently to all aspects of patient care, something interns do not feel well prepared to do. Improved preparedness can be achieved by ensuring curricular outcomes, learning opportunities and assessments are aligned with expectations of internship, enhancing the wellbeing of future graduates as they make the challenging transition to practice.

## Electronic supplementary material

Below is the link to the electronic supplementary material.

Supplementary Material 1

## Data Availability

The data that support the findings of this study are not openly available due to reasons of sensitivity and are available from the corresponding author upon reasonable request.
