# Supplementary material for: Not ready in the ways that count– a qualitative exploration of junior doctor’s perceived preparedness for practice using Legitimation Code Theory
Source: Adv Health Sci Educ Theory Pract. 2024 Oct 7;30(3):795–814. doi: 10.1007/s10459-024-10380-w (PMC12119643; doi:10.1007/s10459-024-10380-w)
Supplement: Supplementary file 1 — Supplementary Material 1 [file 10459_2024_10380_MOESM1_ESM.docx]

**Annexure A - Focus Group Schedule**

Introduction:

Informed consent, privacy and confidentiality (as per the information and consent form) and request to record the session.

Topic for discussion – participants experience and perceptions of being an intern in the South African clinical context.

Questions:

- How would you describe the role of an intern?
- What makes a good intern?
- What are the competencies you need to successfully do your job?
  - Knowledge/Skills/Attitudes
- What attributes are most valued by supervisors?
  - May need clarification
- What signals what is valuable? (from supervisors)
- How ready were you for the role? Why is that?
- What were the things you found most challenging about being an intern?
- In what ways could you have been better prepared? What were your gaps?
- What was competencies/attributes were valued by your medical school when you were a student and how was this signalled?
- What advice would you give current medical students to allow them to be better prepared?
- What advice would you give curriculum developers to makes sure graduates are ready for internship?

Conclusion:

- Moderator summary
- Ask for reflection on the discussion and final comments
- Final question: Have we missed anything?

Thanks
